# Environmental risk factors for reduced kidney function due to undetermined cause in India: an environmental epidemiologic analysis

**DOI:** 10.1097/EE9.0000000000000170

**Published:** 2021-09-24

**Authors:** Sophie A. Hamilton, Prashant Jarhyan, Daniela Fecht, Nikhil Srinivasapura Venkateshmurthy, Neil Pearce, Kabayam M. Venkat Narayan, Mohammed K. Ali, Viswanathan Mohan, Nikhil Tandon, Dorairaj Prabhakaran, Sailesh Mohan

**Affiliations:** a Department of Epidemiology and Biostatistics, MRC Centre for Environment and Health, School of Public Health, Imperial College London, London, United Kingdom; b Public Health Foundation of India, New Delhi, India; c MRC Centre for Environment and Health, School of Public Health, Imperial College London, London, United Kingdom; d Department of Medical Statistics, London School of Hygiene and Tropical Medicine, London, United Kingdom; e Centre for Global NCDs, London School of Hygiene and Tropical Medicine, London, United Kingdom; f Rollins School of Public Health, Emory University, Atlanta; g Madras Diabetes Research Foundation, Chennai, India; h All India Institute of Medical Sciences, New Delhi, India

**Keywords:** Epidemiology, Environmental exposure, India, Chronic kidney disease, Satellite imagery

## Abstract

**Background:**

An epidemic of chronic kidney disease is occurring in rural communities in low-income and middle-income countries that do not share common kidney disease risk factors such as diabetes and hypertension. This chronic kidney disease of unknown etiology occurs primarily in agricultural communities in Central America and South Asia. Consequently, environmental risk factors including heat stress, heavy metals exposure, and low altitude have been hypothesized as risk factors. We conducted an environmental epidemiological analysis investigating these exposures in India which reports the disease.

**Methods:**

We used a random sample population in rural and urban sites in Northern and Southern India in 2010, 2011, and 2014 (n = 11,119). We investigated associations of the heat index, altitude, and vicinity to cropland with estimated glomerular filtration rate (eGFR) using satellite-derived data assigned to residential coordinates. We modeled these exposures with eGFR using logistic regression to estimate the risk of low eGFR, and linear mixed models (LMMs) to analyze site-specific eGFR-environment associations.

**Results:**

Being over 55 years of age, male, and living in proximity to cropland was associated with increased risk of low eGFR [odds ratio (OR) (95% confidence interval (CI) = 2.24 (1.43, 3.56), 2.32 (1.39, 3.88), and 1.47 (1.16, 2.36)], respectively. In LMMs, vicinity to cropland was associated with low eGFR [−0.80 (−0.44, −0.14)]. No associations were observed with temperature or altitude.

**Conclusions:**

Older age, being male, and living in proximity to cropland were negatively associated with eGFR. These analyses are important in identifying subcommunities at higher risk and can help direct future environmental investigations.

## Abstract 2

Supplemental Digital Content is available in the text.

**What this study adds**

Using high-resolution satellite-derived imagery, we modeled key postulated chronic kidney disease of unknown etiology (CKDu) risk factors heat index, altitude, and proximity to land cover class in a large population sample across India. Results show that CKDu is most likely linked to proximity to cropland which could be indicative of pesticide exposure. This is the first study of its kind in India and could be a key first step in identifying specific subcommunities which may be at a higher risk of this disease.

## Introduction

An epidemic of chronic kidney disease is occurring in rural communities in an increasing number of low-income and middle-income countries.^1,2^ Characterized by chronic impairment of kidney function, this disease does not involve known risk factors such as diabetes, hypertension, or proteinuria, and occurs primarily in communities characterized by a hot climate with reliance on heavy agricultural work.^2^ This disease has been termed chronic kidney disease of unknown etiology (CKDu), and is estimated to have led to the premature deaths of hundreds of thousands of young men and women over the past 2 decades.^1^ CKDu is currently defined as having an estimated glomerular filtration rate (eGFR) <60 ml/min/1.73 m^2^ in the absence of diabetes, hypertension, old age (>65 years), or proteinuria.^1–3^

Currently, some of the highest prevalence rates of CKDu have been reported in Uddanam, Southern India and Nicaragua, Central America, where, respectively, 73% and 10%–20% of the adult population sampled in rural areas are affected.^4–6^ Although the etiology of CKDu remains unidentified, there is evidence suggesting that exposure to certain environmental conditions may lead to the development of the disease. Key among these purported environmental risk factors are as follows: (1) high ambient temperatures,^7–11^ (2) physically stressful working environments,^12,13^ (3) low altitude,^14,15^ (4) exposure to pesticides via agricultural farming practices,^4,16–18^ and (5) exposure to heavy metals and other contaminants via potable water sources.^19–22^

Exposure to extreme ambient temperatures can cause dehydration and kidney volume loss, resulting in mortality from exacerbations of an existing chronic disease.^9,23^ Studies investigating the heat hypothesis in relation to CKDu have shown that recurrent heat exposure together with extreme physical exertion and inadequate rehydration can lead to CKD in the absence of common risk factors diabetes, hypertension, or glomerulonephritis.^23,24^ Furthermore, toxic agents^25–27^ in soil and water can spread to wider communities via displacement or absorption into the food chain and can adversely affect susceptible individuals, particularly those with unhealthy lifestyles (i.e., heavy drinkers and/or smokers) and harsh working conditions (long hours conducting high-intensity labor).^18,28^ Altitude is hypothesized to have a protective effect against reduced eGFR^10,14^ with the hormone Erythropoietin thought to help slow renal disease progression, as it attenuates interstitial fibrosis and reduces apoptotic cell death, which is known to be a major contributing factor to the loss of renal function.^29^ It has been postulated that the altitude threshold range for protection against renal damage can be observed between 250 and 1500 m. This estimate; however, this was from a single study, and this association will require further investigation.^14^

Although most cases of CKDu have been reported across Central American countries, there is growing evidence that this disease is also present in India, where the prevalence is estimated to be as high as 73% in selected southern rural communities.^6^ To date; however, no multifactorial environmental exposure studies have been conducted in India, and therefore it is currently unknown whether these risk factors are indeed relevant to the disease observed in the community, and whether environmental risk factors are observed across all CKDu-endemic regions are similar. Our study, therefore, aims to conduct exploratory environmental analyses in urban and rural areas across Northern and Southern India, to investigate whether the risk factors of temperature, altitude, and vicinity to agricultural land are associated with a low eGFR.

## Methods

### Study settings and participants

We used cross-sectional data from two population-based studies conducted in India: the “Centre for Cardiometabolic Risk Reduction in South Asia” cohort study (CARRS study)^30^ and the “Implementing a comprehensive diabetes prevention and management program” study (UDAY study).^31^ Both studies collected socioeconomic, anthropometric, and biosample data including household income, body mass index, blood pressure, and serum creatinine measures. Details on study design, participant selection, and variables collected for these studies have been previously described.^30–32^

**Figure 1.**
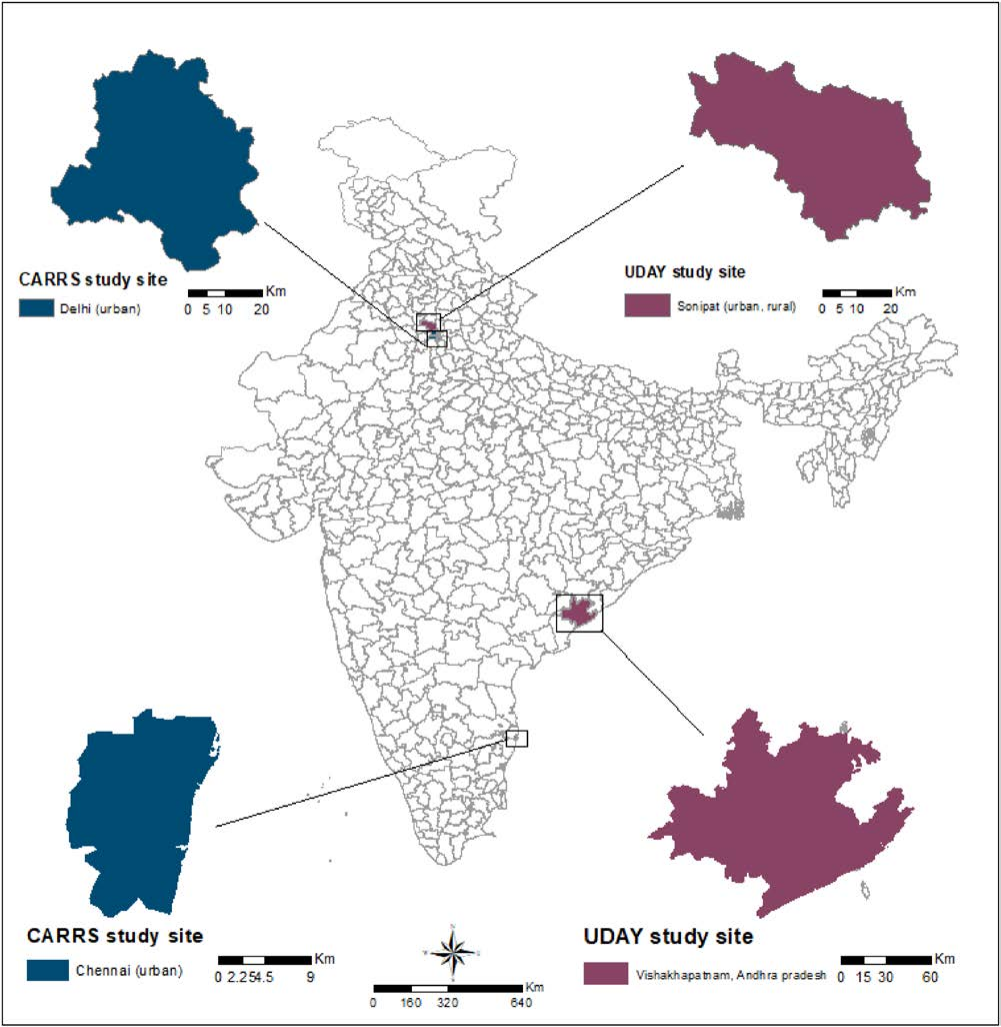
“Centre for Cardiometabolic Risk Reduction in South Asia” (CARRS) and “Implementing a Comprehensive Diabetes Prevention Management Program” (UDAY) study sites located in urban Delhi (Delhi) and rural Chennai (Tamil Nadu) and rural and urban Sonipat (Haryana) and Vishakhapatnam (Andhra Pradesh), respectively.

The CARRS study is a representative sample of adults ≥18 years of age (n = 12,270) between 2010 and 2011 in two urban sites in North (n = 6906) and South (n = 5364) India. The northern site was located in India’s capital city New Delhi which covers a 1483 km^2^ area and has a population of 16,787,941.^33^ The southern site was in Chennai, the capital of Tamil Nadu state which covers an area of 426 km^2^ and has a population of 8,653,521^34^ (Figure 1). We used data from both cross-sectional surveys which comprised 1798 participants from New Delhi and 3193 participants from Chennai.

The UDAY study was conducted on adults ≥30 years in urban and rural sites in Sonipat district, Haryana, North India, and Visakhapatnam district in Andhra Pradesh in South India. In both districts, the program was implemented in a sample of 100,000 participants in each rural and urban subsite with a total population of 400,000 participants. We used data from the first cross-sectional survey conducted among the general population (n= 12,243; Sonipat: n = 6208; Vizag: n = 6035) between July and December 2014. Bio samples were collected from 10,452 participants (Sonipat: n = 5110; Vizag: n = 5342). In Sonipat, the urban site was in Sonipat city (n = 3104), which covers a 388-km^2^ area, and has a population of 1,450,000.^33^ The rural site was in the Kharkhoda subdistrict (n = 3104) which covers 278 km^2^ with a population of 135,844^33^ (Figure 1). The southern sites were located in Andhra Pradesh on the South East coast in the Visakhapatnam district (Vizag) which covers 11,161 km^2^, with a population of 4,290,589.^33^ The urban site was in the city of Visakhapatnam (n = 2966), and the rural site was in mandals (which are similar to an administrative area) Makavarapalem and Nathavaram (n = 3069) (Figure 1).^31^ The dataset comprised 1540 participants from urban Sonipat, 1640 participants from rural Sonipat, 1228 participants from urban Visakhapatnam, and 1816 participants from rural Visakhapatnam were included in the analysis.

We investigated the associations of the following environmental risk factors: (1) ambient temperature,^23,35^ (2) altitude,^10,36^ and (3) residential proximity to agricultural land^37^ with eGFR as a continuous variable and risk of eGFR <60 as a categorical marker of CKD stage 3 or worse.^1,38^ Proximity to agricultural land is increasingly being studied as a proxy to agrochemical exposure due to resource and financial constraints of effectively measuring pesticides in laboratories. Using satellite-derived environmental data, we assigned these environmental exposures to participant residential coordinates which were collected over the CARRS and UDAY study periods.^30,31^

#### Data sources

We used satellite-derived heat index (HI), altitude, and land cover data to estimate participants’ exposure.

To estimate the combined effects of temperature and humidity on the human body, we used an HI.^39–42^ The HI is a commonly used exposure proxy for heat stress in environmental health studies^43–45^ as it provides a “feels like” heat measure and is considered preferable to measuring air temperature alone.^41,42^ We used the retrospective ERA-5 Land re-analysis dataset from the European Centre for Medium-Range Weather Forecasts which has a spatial resolution of 9 km.^46^ We extracted monthly averaged 2-m height temperature and 2-m height dew point variables between October 2010–November 2011 and July 2014–December 2014 to capture the timeframes across which the CARRS and UDAY studies were conducted, respectively.

First, using the R studio “Weathermetrics” package we calculated the relative humidity using the temperature and dew point data variables using the following equation:


$$R=100*\left\{\frac{\text{EXP}\;\;\left(\left(17.625*T_{\text{d}})/(243.04+T_{\text{d}}\right)\right)}{\text{EXP}\;\;\left(\left(17.625*T)/(243.04+T\right)\right)}\right\}$$

where *R* = relative humidity, *T*_d_ = Dewpoint, *T* = Temperature.^47^

We then calculated the HI for each grid point in Celsius units using the following equation:


$$\begin{array}{l} \text{HI}=-42.379+2.04901523T+10.14333127R-0.22475541TR\\ \;\;\;\;\;\;\;\;\;\;\;\;\;\;\;\;\;\;\;\;\;\;\;\;\;\;\;-6.83783\times 10-3T2\;\\ \;\;-5.481717\times 10-2R2+1.22874\times 10-3T2R+8.5282\times 10-4TR2\\ \;\;\;–\;1.99\times 10-6T2R2 \end{array}$$

where HI = heat index; *T* = ambient temperature; *R* = relative humidity.^48^

Using ArcGIS software, we modeled a continuous HI surface across the study sites, using the probabilistic interpolation method Kriging. For geostatistical modeling, the structure of spatial variation is estimated through the semivariogram which is a visual depiction of the covariance exhibited between each pair of points in the sampled data and determines the weights to apply to data points when developing predictions.^49^

The variogram is expressed as:


$$\begin{array}{c} \gamma (h)=\frac{1}{2}\text{ Var}\;\;\;\{\left(Z(s)-\mu (s)]-[Z(s+h)-\mu (s+h)\right)\} \end{array}$$

where *h* denotes the translation between any two sites s_*i*_ and s_*j*_ in a study area.^50^

We conducted a sensitivity analysis comparing the Kriging method with other interpolation methods inverse distance weighting and Empirical Bayesian Kriging (EBK) to assess the best overall fit. We used a holdout cross-validation approach by which the dataset is randomly divided into a training and validation set (Table S1, Supplemental Digital Content 1; http://links.lww.com/EE/A153). The model is trained on the training dataset and evaluated on the validation dataset.^51^ The overall fit was evaluated on the basis of the root mean squared error (RMSE) and the mean actual error. Sensitivity analyses showed that Kriging was the most accurate interpolation method in comparison to inverse distance weighting and EBK (Tables S2–S5, Supplemental Digital Content 1; http://links.lww.com/EE/A153). The prediction errors were the lowest across Delhi (Table S3, Supplemental Digital Content 1; http://links.lww.com/EE/A153), Haryana (Table S5, Supplemental Digital Content 1; http://links.lww.com/EE/A153), and Tamil Nadu (Table S4, Supplemental Digital Content 1; http://links.lww.com/EE/A153) (RMSE = 0.006, 0.010, and 0.010, respectively). Higher prediction errors were observed in Andhra Pradesh (RMSE = 0.085) (Table S2, Supplemental Digital Content 1; http://links.lww.com/EE/A153).

To create a continuous altitude surface across the study sites, we used a digital elevation model (DEM) from the Shuttle Radar Topography Mission, a global DEM giving coverage of void-filled data at a resolution of 30 m with a vertical accuracy of 20 m.^52^ Altitude values were then assigned to the participant residential coordinate.

For land cover, we used The European Space Agency Climate Change Initiative programme raster products which have a 300-m resolution^53^ and a typology comprising 22 classes^53^ including cropland and urban cover. To capture and assign a land cover class in the immediate neighborhood of each participant, we placed a 300-m buffer around each residential coordinate to match the land cover data resolution. We assigned the mode land cover class inside each buffer to capture the most common class for each participant and then grouped classes into “cropland” and “urban” cover classes. All environmental exposure values were assigned to residential coordinates using ArcGIS software version 10.5.1 by ESRI.

### Data cleaning and coding

The dataset was pre-restricted for those with missing serum creatinine, age, and sex variables, as were those diagnosed or self-reported with diabetes (fasting plasma glucose ≥126 mg/dl), hypertension (systolic blood pressure ≥140 mm Hg, or diastolic blood pressure ≥90 mm Hg) and proteinuria [albumin/creatinine ratio (ACR) in urine ≥300 mg/g]. Detail on how these variables were measured are described elsewhere.^30,31^ We assigned participant household coordinates to the restricted dataset using participant ID numbers. For more parsimonious models, we re-grouped income categories into three groups “unknown,” “<30,000RS,” and “≥ 30,000RS” which represented the mid-point of the salary categories. Those with missing coordinate data were also excluded.

Kidney function is measured using the eGFR calculated using serum creatinine, age, and sex variables.^38^ Normal kidney function is defined by an eGFR >90 ml/min/1.73 m^2^ body mass area.^54^ For this dataset, eGFR was calculated using the Chronic Kidney Disease-Epidemiology Collaboration equation using serum creatinine, age, and sex data.^38^

### Statistical analyses

We used linear regression models to estimate the associations between eGFR and socioeconomic, anthropometric, and environmental exposure variables; and logistic regression models to estimate odds ratios (ORs) and corresponding 95% confidence intervals (CI) for risk of having a low eGFR (<60 ml/min/1.73 m^2^) in relation to the aforementioned variables.

We tested each linear and logistic regression model for multicollinearity using the variance inflation factor (VIF) >10 as the threshold for exclusion. Due to multicollinearity between HI and altitude, we modeled these variables separately in the linear and logistic regression models.

Having ascertained the key risk factors for low eGFR and eGFR <60, we assessed the effects of environmental exposures on eGFR alone, accounting for different geographical locations. To do this, we used Linear mixed models (LMMs) to model associations between eGFR and environmental exposures between different geographical locations, adjusting for age and sex. LMMs are an extension of simple linear models which allow both fixed and random effects and are used when there is nonindependence in the data. The model assumes that observations in the same cluster (or study site) are more correlated than those in another study site,^55^ that the average eGFR will differ between sites, and that the effect of environmental exposures on eGFR is stable across the study site. We modeled associations between environmental exposures HI, altitude, and land cover with eGFR. In each model, the fixed effects were eGFR and the satellite-derived environmental exposures listed above, and the random structures were stated with the subcategory “urban/rural area” which represented each study site. We then calculated a fixed-effects model which illustrates how each environmental exposure affects eGFR across the study sites.

All statistical analyses were conducted in R Studio version 3.5.1.

## Results

### Study population characteristics

**Figure 2.**
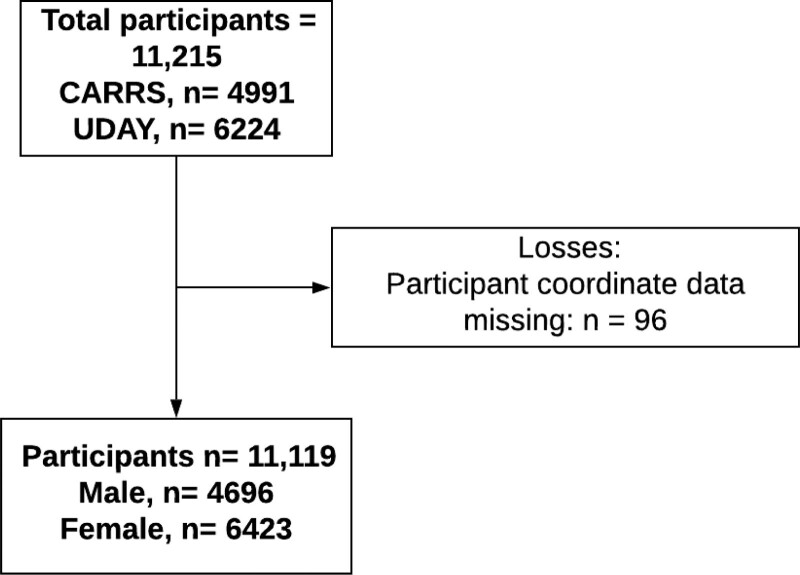
Study flowchart with exclusion criteria for the India population sample. From the original, prerestricted CARRS and UDAY datasets, one transgender participant was removed. Missing data: Serum creatinine n = 3960; diabetes = 209; hypertension = 517; missing albumin:creatinine ratio (ACR) = 735. Participants with CKD risk factors removed: Diabetic = 4203; hypertensive = 2468; ACR >300 = 203.

The study population comprised 11,119 adult participants ≥18 years (males = 4696; females = 6423) who did not have diabetes (fasting glucose <126 mg/dl), hypertension (systolic <140 mm Hg, and diastolic <90 mm Hg), or proteinuria (ACR < 30 mg/mmol) as per diagnostic cutoffs defined in the existing CKDu measurement protocol which is described elsewhere.^1^ Participant with missing coordinates was excluded from the dataset (n = 96) (Figure 2). The mean (±SD) participant age was 41.5 (±11.7) years. Mean BMI was 23.6 ± 5.3 kg/m^2^, and mean fat-free mass was 41.0 ± 13.0 kg. Mean systolic and diastolic blood pressure was 114.2 ± 11.7 mm Hg, and 73.2 ± 8.7 mm Hg, respectively. Mean fasting plasma glucose was 90.2 ± 12.4 mg/dl, and the median (interquartile range ACR was 2.6 (1.0–5.0) mg/mmol. Approximately 50% of the participants were employed, and 41% of the population had completed >10 years of formal education.

**Figure 3.**
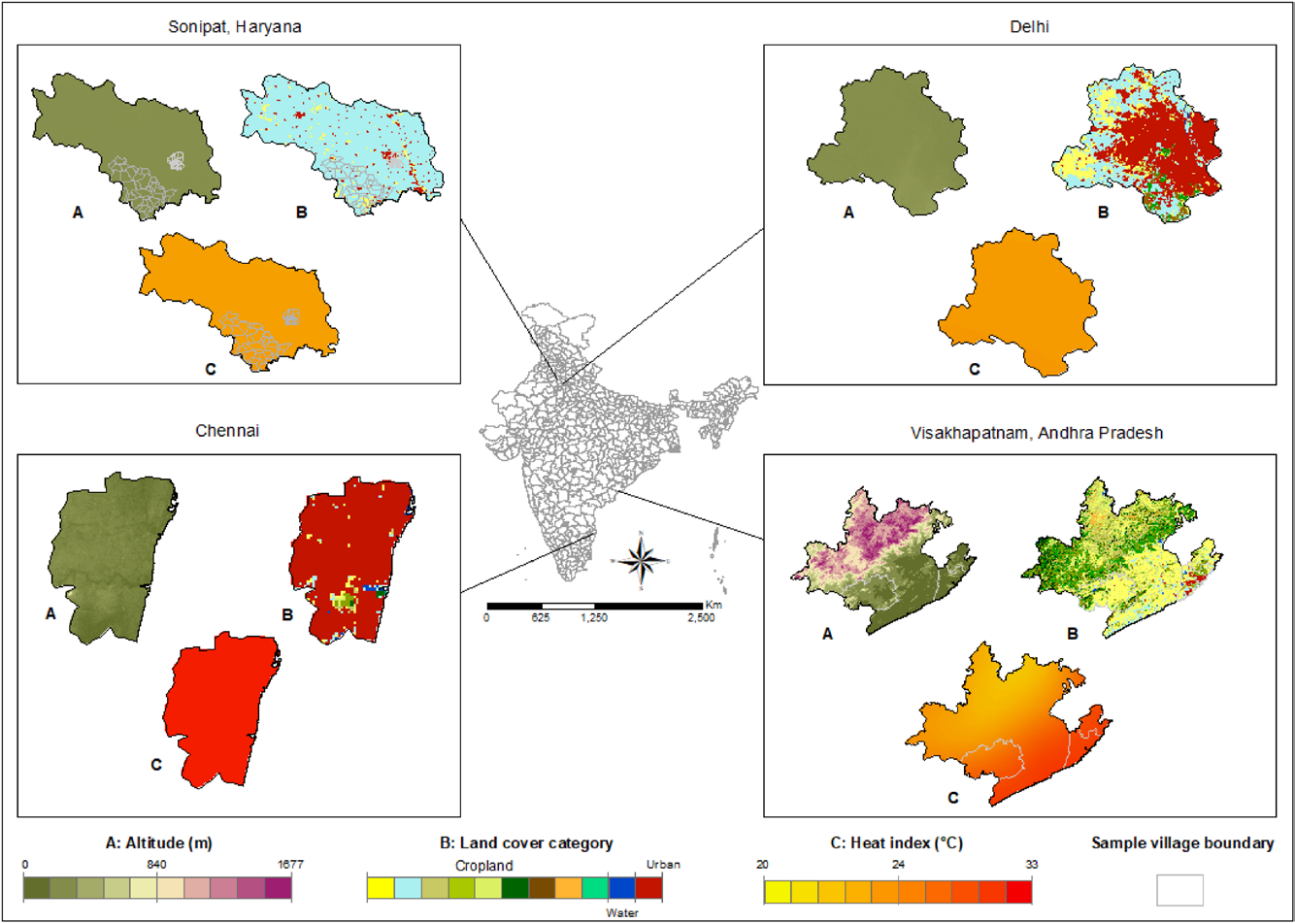
Environmental variable surfaces across study sites showing (A) altitude, (B) land cover, and (C) heat index.

The HI values assigned to participants ranged from 23.95 to 30.31°C (Figure 3C), and approximately 45% of participants lived within 300 m of “cropland” (n = 4951), 70% of which lived in rural areas (n = 3449) (Figure 3B). The altitude ranged from 1 to 391 m above sea level (Figure 3A). See Table 1 for a summary of environmental characteristics per study site.

**Table 1.** Overview of environmental characterisitcs of the Indian study sites.

Site	n	Mean age (±SD)	Sex		Latitude	eGFR (mL/min/1.73 m^2^)	CKDu prevalence (%)	Heat index (°C)	Altitude (m)	Land cover (%)	
			Male	Female						Urban	Cropland
Haryana	3180	38.93 (12.01)	1340	1840	North	101.81	1.4	25.27–25.58	206–247	38.5	61.5
Delhi	1798	38.93 (11.14)	819	979	North	110.23	0.8	23.95–24.34	1–292	65.6	34.4
Tamil Nadu	3097	36.87 (10.85)	1202	1895	South	114.13	0.3	30.20–30.31	1–17	87.1	12.9
Andhra Pradesh	3044	43.15 (10.69)	1335	1709	South	99.49	3.2	25.80–28.31	0–391	35.0	65.0

### Mean eGFR and prevalence of low eGFR

The mean eGFR was 105.9 (±17.5) ml/min/1.73 m,^2^ and there was an inverse relationship between increasing age, FFM, altitude, and income. Males, rural dwellers, those in the vicinity to “cropland” and alcohol drinkers (ever) and/or smokers had a lower eGFR. The prevalence of eGFR <60 was 1.4% [95% confidence interval (95% CI) = 1.2, 1.7]. A significant difference in sex-specific prevalence of eGFR <60 was observed, with 2.0% (95% CI = 1.7, 2.5) of males versus 1.0% (95% CI = 0.8, 1.3) of females affected. Site-specifically, the highest prevalence of eGFR <60 was observed in Andhra Pradesh [3.2% (95% CI = 2.5, 3.8)].

### Risk factors for reduced eGFR and eGFR <60

Using linear regression models, we tested for multicollinearity between altitude and HI, as these are generally inversely correlated. In fully adjusted models we observed multicollinearity between altitude (VIF 25.06) and HI, and north/south latitude with HI (VIF = 12.06). HI can be used as a proxy for latitude, as broadly temperatures in the north of India are generally cooler than in the South.^56^ Tables 2 and 3 show linear and logistic regression models including crude estimates, models mutually adjusted for age and sex (model 1), and models fully adjusted for all socioeconomic and environmental risk factor variables (model 2). We observed large decreases in effect estimates between crude and minimally adjusted linear regression models specifically for sex, proximity to cropland, and education. After further stratified analyses, age was the key driver of these changes. In general, male participants were older than females and had a lower eGFR, and a higher proportion of older participants (with a lower eGFR) live in proximity to cropland than those in urban areas. In the case of education, an increasing number of school years were inversely associated with age. These factors likely explain these stepped differences in effect estimates when adjusted for age.

**Table 2.** Associations of sociodemographic, anthropometric, and environmental characteristics with eGFR in participants without diabetes, hypertension, and heavy proteinuria in India, n = 11,119

Variable	Crude effect estimate	Model 1, Minimal adjustment	Model 2, Fully adjusted
	eGFR	eGFR	eGFR
	Coefficient (95% CI)	Coefficient (95% CI)^a^	Coefficient (95% CI)^b^
Age^c^ (10-year increase)	−9.58 (−9.79, −9.37)	−9.38 (−9.59, −9.16)	−9.11 (−9.34, −8.66)
Sex^d^			
Male	−6.67 (−7.32, −6.02)	−3.70 (−4.20, −3.19)	−2.46 (−3.19, −1.73)
Female		REF	REF
Education (years)			
≤5	REF	REF	REF
>5≤10	7.29 (6.45, 8.13)	2.20 (1.56, 2.88)	1.27 (0.60, 1.95)
>10	6.10 (5.24, 6.78)	0.71 (0.98, 1.33)	0.18 (−0.48, 0.84)
Occupation			
Employed	REF	REF	REF
Unemployed	2.54 (1.89, 3.19)	2.24 (1.59, 2.89)	1.68 (1.03, 2.34)
Household monthly income (RS)^e^			
≤30,000	4.56 (4.43, 6.90)	2.96 (2.01, 3.91)	2.21 (1.23, 3.19)
>30,000	REF	REF	REF
Unknown	−1.28 (−3.65, 1.09)	−1.04 (−3.01, 0.92)	−1.03 (−2.8, 0.77)
BMI (kg/m^2^) 5 kg/m^2^ increase	−0.49 (−0.72, −0.27)	−0.58 (−0.79, −0.37)	−0.60 (−0.82, −0.38)
Fat-Free Mass (kg) 5 kg/m^2^ increase	−0.63 (−0.73, −0.52)	−0.24 (−0.34, −0.15)	−0.16 (−0.26, −0.05)
Smoker			
Yes	−1.44 (−2,19, −0.69)	−0.30 (−0.87, 0.27)	−0.35 (−1.00, 0.28)
No	REF	REF	REF
Alcohol drinker			
Yes	−1.13 (−1.93, −0.33)	0.28 (−0.32, 0.89)	0.09 (−0.59, 0.78)
No	REF		REF
Vegetarian			
Yes	2.61 (−5.30, 3.92)	0.25 (−0.29, 0.79)	1.73 (1.11, 2.35)
No	REF	REF	REF
Heat index (°C)			
0.2 increments	0.28 (0.25, 0.31)	0.23 (0.18, 0.28)	0.20 (0.05, 0.10)
Land cover			
Cropland	−7.82 (−8.46, −7.18)	−3.40 (−3.90, −2.89)	−2.83 (−3.36, −2.31)
Urban	REF	REF	REF
Altitude*			
100 m increments	−2.25 (−2.58, −1.92)	−0.03 (−0.14, 0.11)	−0.04 (−0.09, 0.22)

^a^Minimal adjustment for age, sex.

^b^All variables mutually adjusted.

^c^Adjusted for sex.

^d^Adjusted for age.

^e^Exchange rate (RS to USD) 0.001 at time of questionnaire; Hypertension = systolic bp ≥140 mm Hg, or diastolic bp ≥90 mm Hg; Diabetes = fasting glucose ≥7 mg/l; Proteinuria = ACR [Albumin Creatinine Ratio] ≥30 mg/mmol.

*Effect estimate for altitude modeled separately from heat index due to multicollinearity.

**Table 3.** Associations of sociodemographic, anthropometric, and environmental characteristics with eGFR <60 in participants without diabetes, hypertension, and heavy proteinuria in India, n = 11,119.

Variable	Crude effect estimate	Model 1, Minimal adjustment	Model 2, Fully adjusted
	eGFR < 60	eGFR < 60	eGFR < 60
	OR (95% CI)	OR (95% CI)^a^	OR (95% CI)^b^
Age (years)^c^			
18–24	0.17 (0.01, 0.83)	0.17 (0.01, 0.83)	0.32 (0.02, 1.60)
25–35	0.16 (0.07, 0.34)	0.18 (0.08, 0.36)	0.24 (0.10, 0.51)
36–45	0.32 (0.18, 0.55)	0.32 (0.19, 0.56)	0.38 (0.22, 0.66)
46–55	REF	REF	REF
56–65	2.52 (1.63, 3.96)	2.51 (1.61, 3.94)	2.24 (1.43, 3.56)
65 inf	6.77 (4.42, 10.51)	4.88 (4.15, 9.92)	4.71 (2.90, 7.72)
Sex^d^			
Male	2.02 (1.48, 2.78)	1.44 (1.05, 1.99)	2.32 (1.39, 3.88)
Female	REF	REF	REF
Education (years)			
≤5	REF	REF	REF
>5≤10	0.32 (0.22, 0.48)	0.47 (0.32, 0.77)	0.54 (0.36, 0.83)
>10	0.16 (0.09, 0.23)	0.22 (0.14, 0.35)	0.25 (0.14, 0.43)
Occupation			
Employed	REF	REF	REF
Unemployed	1.15 (0.85, 1.57)	0.93 (0.63, 1.39)	1.34 (0.89, 2.03)
Household monthly income (RS)^e^			
≤30,000	1.24 (0.69, 2.52)	1.35 (0.74, 2.77)	0.48 (0.24, 1.05)
>30,000	REF	REF	REF
Unknown	1.81 (0.61, 4.94)	1.53 (0.51, 4.26)	0.40 (0.13, 1.21)
Body Mass Index (kg/m^2^) Underweight (≤18.5)	1.13 (0.74, 1.68)	0.79 (0.30, 0.78)	0.59 (0.38, 0.93)
Normal (>18.5–≤25)	REF	REF	REF
Overweight (>25–≤30)	0.39 (0.24, 0.61)	0.49 (0.30, 0.78)	0.66 (0.40, 1.06)
Obese (>30)	0.39 (0.06, 0.49)	0.28 (0.10, 0.70)	0.44 (0.13, 1.11)
Fat-Free Mass (kg) First tertile (≤37)	1.42 (0.94, 2.14)	2.13 (1.32, 3.48)	1.38 (0.76, 2.54)
Second tertile (>37–<45)	1.22 (0.80, 1.88)	1.52 (0.97, 2.40)	1.32 (0.70, 1.83)
Third tertile (≥45)	REF	REF	REF
Smoker			
Yes	1.35 (0.96, 1.87)	1.15 (0.82, 1.61)	1.11 (0.75, 1.65)
No	REF	REF	REF
Alcohol drinker			
Yes	1.24 (0.86, 1.76)	1.10 (0.76, 1.57)	1.07 (0.70, 1.63)
No			REF
Vegetarian			
Yes	0.84 (0.60, 1.18)	0.54 (0.38, 0.76)	0.81 (0.51 1.31)
No	REF	REF	REF
Heat index (°C)			
<26	REF	REF	REF
>26	1.26 (0.92, 1.73)	1.02 (1.02, 2.44)	0.37 (0.95, 2.26)
Land cover			
Cropland	2.82 (2.04, 3.97)	1.88 (1.35, 2.67)	1.47 (1.16 2.36)
Urban	REF	REF	REF
Altitude (m)*			
<100	1.05 (0.77, 1.44)	1.51 (0.81, 2.08)	1.28 (0.87, 1.91)
>100	REF	REF	REF

^a^Minimal adjustment for age, sex.

^b^All variables mutually adjusted.

^c^Adjusted for sex.

^d^Adjusted for age.

^e^Exchange rate (RS to USD) 0.001 at time of questionnaire; Hypertension = systolic bp ≥140 mm Hg, or diastolic bp ≥90 mm Hg; Diabetes = fasting glucose ≥7 mg/l; Proteinuria = ACR [Albumin Creatinine Ratio] ≥30 mg/mmol.

*Effect estimate for altitude modeled separately from heat index due to multicollinearity.

Overall, the coefficients do not vary widely between minimally and fully adjusted models and we report the results from the fully adjusted linear and logistic regression models only (Tables 2 and 3, model 2, respectively).

In linear regression models (Table 2, model 2), age was a key risk factor for reduced eGFR, with a decrease of 9.11 ml/min/1.73 m^2^ (95% CI = −9.34, −8.66) per 10-year age increase. Males also had a lower average eGFR than females [−2.46, (95% CI) = (−3.19, −1.73)]. Positive associations were observed with vegetarianism, education (>5 ≤10 years), and lower income (<30,000 RS). For the environmental exposures, living near cropland [−2.83 (95% CI) = (−3.36, −2.31)] had a negative effect on eGFR. Interestingly, increasing HI had a weak positive association with eGFR [(0.20 (95% CI = 0.05, 0.10)].

Like the linear regression model, the odds of eGFR <60 increased with age, particularly in the older categories 56–65, and over 65 years [OR (95% CI) = 2.24 (1.43, 3.56) and 4.71 (2.90, 7.72), respectively], being male [OR (95% CI) = 2.32 (1.39, 3.88)] and living in proximity to cropland [OR (95% CI) = 1.47 (1.16, 2.36)]. A marginal protective effect was observed with years of education and vegetarianism (Table 3, model 2).

### Linear mixed models

**Figure 4.**
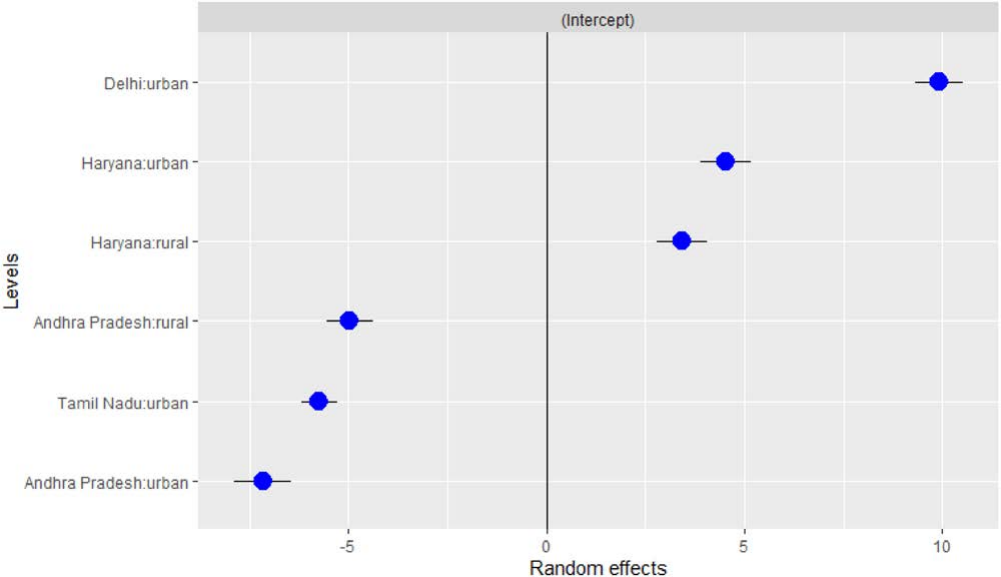
Linear mixed model caterpillar plot of eGFR in study sites in India accounting for land cover, heat index, and altitude. Intercept denotes the overall mean eGFR across the study sites; blue circles represent the deviation from the mean eGFR for each zone; black bars represent 95% confidence intervals.

Figure 4 shows the results of the LMM for India which has been modeled with a hierarchy of state and urban/rural area as the random structures, and environmental exposures HI, altitude, and cropland as the fixed effects. The results indicate that the Southern study sites in Tamil Nadu and Andhra Pradesh had the lowest ranking mean eGFR values, and the northern urban sites in Delhi and Haryana had the highest. The mean eGFR values in rural Haryana (northern India) did not deviate from the overall population sample mean. The fixed-effects model (Table 4) shows the effects of the environmental exposures, HI, altitude, and cropland on eGFR in each study site, adjusted for age and sex.

**Table 4.** Linear mixed model for altitude, heat index, and land cover in India.

Variable	Regression coefficient	Confidence interval (95%)
Age (10-year increments)	−8.68	−9.89, −1.84
Sex		
Male	−3.70	−4.19, −3.21
Female	REF	REF
Heat index (°C) (0.2°C increments)	0.53	0.89, 1.14
Land cover		
Cropland	−0.80	−0.44, −0.14
Urban	REF	REF
Altitude (m) (100 m increments)*	−1.13	−2.56, 0.09

*Effect estimate for altitude modeled separately from heat index due to multicollinearity.

Residential proximity to cropland had a small negative association with mean eGFR [−0.89 (−0.44, −0.14)]. A weak protective association was observed with increasing temperatures [0.53 (0.89, 1.14)].

## Discussion

Our environmental epidemiological analysis was conducted in states across Northern and Southern India; CKDu is known to be endemic in the latter region. We used data from 11,119 adult participants to assess whether key hypothesized environmental exposures such as HI, altitude, and proximity to land cover type were associated with having an eGFR <60 ml/min/1.73 m^2^ in the absence of diabetes, hypertension, or proteinuria. Across all study sites, increasing age and sex (males) were associated with both low eGFR and increased risk of eGFR <60.

The environmental conditions observed across this population (higher temperatures, lower altitude, and much of the population living in and around agricultural land) concur with the environmental characteristics associated with CKDu across the literature.^13,37,57,58^ The highest prevalence of CKDu was observed in Andhra Pradesh. This observation also corroborates with findings from other Indian studies^6,59^ which found that the prevalence of CKDu was markedly higher in this region in comparison with other urban and rural sites.

Of the environmental risk factors, vicinity to cropland was associated with both low eGFR and risk of eGFR <60. This finding corroborates with a previous study in El Salvador whereby proximity to specific crop types was significantly associated with suspected CKDu mortality and hospital admissions rates.^37^ There are few studies that investigated the spatial distribution of CKDu in relation to proximity to land cover; however, there are some studies and reviews in India which investigated the association between CKDu and agricultural occupation, which suggest that those cultivating rice and cashew crops, as well as those using pesticides are the most affected subgroup of agricultural laborers.^16,59,60^ Although the current dataset does not contain detailed employment information or high-resolution crop detail, collecting this data will be key in future studies across India to geographically highlight specific subcommunities which may be at a higher risk and help direct future observational and treatment plans in affected regions.

The LMM results presented slightly different observations from the linear and logistic regression models. The southern Indian study sites in Tamil Nadu and Andhra Pradesh had the lowest ranking mean eGFR values, and the northern urban sites in Delhi and Haryana had the highest. Like the linear and logistic regression models, vicinity to cropland had a negative association with eGFR.

Interestingly, a weak positive association was observed with increasing HI and eGFR. This observation does not support the hypothesis of heat stress nephropathy that persistent heat exposure and inadequate rehydration leading to CKD and may therefore count against the current heat hypothesis.^9,12,23^ Although exposure to high temperatures is a key hypothesis in the literature, there are some important counter arguments that are important to consider. Herath et al.^61^ argued that there is sparse evidence of CKDu in workers exposed to heat in most tropical regions, which was supported by the finding that it is also seen in people who are not exposed to heat stress in these affected regions, and therefore there is inadequate evidence for heat being the initiating or main cause of CKDu. In addition to this, a systematic review of risk factors for CKDu in Central America did not identify heat as a risk factor.^3^

Our study has some potential limitations. First, our dataset had only single eGFR measures meaning we may not distinguish acute kidney injury from CKD, potentially resulting in case misclassification and inflated prevalence estimates. Furthermore, the use of cross-sectional data cannot prove causality in relation to environmental exposures; however, it can help to generate causal hypotheses which can be further investigated in future cohort studies in affected regions. Second, there were no occupational variables in our dataset, therefore it was not possible to link the proximity to landcover associations to a specific occupational category in this population. In addition, the use of indirect measures of exposure to pesticides could have resulted in exposure misclassification potentially attenuating estimates.

Finally, unmeasured biological confounders across this Indian sample – such as endemic hepatitis which is linked with nephropathy^62–64^ – could be linked to excess CKD prevalence was not measured in these populations which could also affect our estimates. Further sensitivity analyses into the effects of these residual confounders such as probabilistic bias analysis^65^ could be investigated in future work; however, this is outside the scope if this study.

Strengths of our study include the use of a large, randomly selected sample population in urban and rural areas across the north and south of India. Second, this is the first study in India that has used satellite-derived imagery to investigate associations between environmental risk factors and CKDu.

The use of satellite measurements has advantages over conventional ground measurements as data can be collected repeatedly and automatically and can provide better coverage than ground monitors. The growth in the use of remote sensing and geographic information systems in public health has facilitated the analyses of multiple environment-disease associations at varying geographical resolutions and continues to be a valuable exposure analysis tool, particularly in developing nations that may be too resource-constrained to conduct individual-level environmental exposure analyses.

## Conclusions

The findings from this environmental epidemiological analysis show that the environmental risk factor of residential proximity to cropland (particularly in Southern India) appears to have a negative impact on eGFR. Although we must be cautious in our interpretation of these initial observations, these findings are inconsistent with the current hypothesis that CKDu is a heat-induced disease but are reasonably consistent with some of the other hypotheses such as exposure to pesticides using proximity to cropland as a proxy. The use of satellite-derived data to model environmental exposures of CKDu at the individual level is a useful step in identifying subpopulations at risk of CKDu and could help to direct further environmental investigations in affected regions. In future studies, the collection of detailed employment information and occupational practices will be key in helping to identify further potential associations with environmental risk factors.

## ACKNOWLEDGMENTS

None.

## Supplementary Material


