# Environmental risk factors for reduced kidney function due to undetermined cause in India: an environmental epidemiologic analysis: Erratum

**DOI:** 10.1097/EE9.0000000000000197

**Published:** 2022-02-07

In the article in the September 2021 compendium, there were some errors.

After publication of this original article, it came to the authors’ attention that the spelling of K.M. Venkat Narayan’s family name and order of initials were incorrect. Additionally, their ORCID number was not added.

K. M Venkat Narayan’s first and second initials and family name have been re-ordered and spelled correctly in this erratum, and their ORCID number has been added: [ORCID 0000-0001-8621-5405]
