# Supplementary material for: Environmental risk factors for reduced kidney function due to undetermined cause in India: an environmental epidemiologic analysis
Source: Environ Epidemiol. 2021 Sep 24;5(5):e170. doi: 10.1097/EE9.0000000000000170 (PMC8683143; doi:10.1097/EE9.0000000000000170)
Supplement: Supplementary file 1 [file ee9-5-e170-s001.pdf]

Appendix I

Table S1. Kriging hold-out validation summary showing model fit for heat index across Indian study sites

**Prediction error measures kriging**	**Efficiency**	**Prediction error**	
Study site	R^2	RMSE	MAE
Delhi	0.962	0.006	0.005
Haryana	0.990	0.010	0.008
Tamil Nadu	0.759	0.010	0.007
Andhra Pradesh	0.990	0.085	0.067

R^2 = coefficient of determination, RMSE = root mean square error, MAE = mean absolute error

Table S2. K-fold cross validation Summary of the performance of interpolation methods to predict heat index across Andhra Pradesh

Interpolation method	Efficiency	Prediction error	
	R^2	RMSE	MAE
IDW	0.991	0.089	0.070
Kriging	0.990	0.085	0.067
EBK	0.906	0.277	0.193

R^2 = coefficient of determination, RMSE = root mean square error, MAE = mean absolute error, IDW: inverse distance weighting; EBK: Empirical Bayesian Kriging

Table S3. K-fold cross validation Summary of the performance of interpolation methods to predict heat index across Delhi

Interpolation method	Efficiency	Error	
	R^2	RMSE	MAE
IDW	0.980	0.004	0.002
Kriging	0.962	0.006	0.005

EBK	0.981	0.007	0.008

R^2 = coefficient of determination, RMSE = root mean square error, MAE = mean absolute error, IDW: inverse distance weighting; EBK: Empirical Bayesian Kriging

Table S4. K-fold cross validation Summary of the performance of interpolation methods to predict heat index across Tamil Nadu

Interpolation method	Efficiency	Error	
	R^2	RMSE	MAE
IDW	0.664	0.016	0.011
Kriging	0.759	0.010	0.007
EBK	0.702	0.024	0.012

R^2 = coefficient of determination, RMSE = root mean square error, MAE = mean absolute error, IDW: inverse distance weighting; EBK: Empirical Bayesian Kriging

Table S5. K-fold cross validation Summary of the performance of interpolation methods to predict heat index across Haryana

Interpolation method	Efficiency	Error	
	R^2	RMSE	MAE
IDW	0.986	0.010	0.008
Kriging	0.990	0.010	0.008
EBK	0.437	0.068	0.050

R^2 = coefficient of determination, RMSE = root mean square error, MAE = mean absolute error, IDW: inverse distance weighting; EBK: Empirical Bayesian Kriging
